# Health-related quality of life of postpartum women and associated factors in Dendi district, West Shoa Zone, Oromia Region, Ethiopia: a community-based cross-sectional study

**DOI:** 10.1186/s12905-024-02918-2

**Published:** 2024-01-31

**Authors:** Iranfachisa Gurmu Amana, Eden Girmaye Tefera, Eshetu Ejeta Chaka, Gizachew Abdissa Bulto

**Affiliations:** 1 Ambo University Referral Hospital, Ambo, Oromia Region Ethiopia; 2 Department of Midwifery, Ambo University, Ambo, Oromia Region Ethiopia; 3 Department of Public Health, Ambo University, Ambo, Oromia Region Ethiopia

**Keywords:** Quality of life, Health-related quality of life, Postpartum women, Maternal health services, Ethiopia

## Abstract

**Background:**

Promoting a favorable experience of postpartum care has become increasingly emphasized over recent years. Despite the fact that maternal health care services have improved over the years, postnatal care service utilization is generally low and the health-related quality of life of postpartum women remains overlooked. Furthermore, the health-related quality of life of postpartum women is not well studied. Therefore, this study aimed to assess the health-related quality of life of postpartum women and associated factors in Dendi district, West Shoa Zone, Oromia, Region, Ethiopia.

**Methods:**

A community-based cross-sectional study was conducted among 429 participants. A multistage stratified sampling procedure was used to select the sampling unit and simple random sampling technique was employed to select the study participants from 23 August 2022 to 16 November 2022. A pre-tested standard structured questionnaire was used to collect the data. Data were entered using Epi-Data 3.1 and then exported to Statistical package for social science version 26. Binary logistic regression analysis was computed at p-value < 0.25 were considered candidates for multivariable logistic regression. Adjusted Odds Ratios (AOR) with 95% confidence interval and statistical significance was declared at a p-value < 0.05.

**Results:**

The study revealed that 73.7% (95% CI: 69.4–77.7) had a low level of health-related quality-of-life with a mean of 44.02 (SD ± 10.4). Urban residing [AOR = 0.27, 95% CI: (0.10–0.74)], no education [AOR = 3.44, 95% CI (1.35–8.74)], received at least four antenatal contact [AOR = 0.56, 95% CI (0.33–0.95)], received at least one postnatal care [AOR = 0.30, 95% CI (0.14–0.62)], poor social support [AOR = 2.23, 95% CI: (1.025–4.893)], having postpartum depression [AOR = 2.99, 95% CI: (1.52–5.56)], cesarean delivery [AOR = 3.18, 95% CI: (1.09–9.26)], and lowest household assets [AOR = 5.68, 95% CI: (2.74–11.76)] were significant associations with low health-related quality of life of postpartum women.

**Conclusions:**

The health-related quality of life among postpartum women was very low. Postpartum women with low socio-economic status and inadequate maternal health service utilization had a low health-related quality of life. Promoting women’s education and postnatal care services is needed to improve the health-related quality of life of postpartum women.

## Introduction

Women experience significant physiological and psychological changes following childbirth [1]. During the postpartum period, women faced difficulties adjusting to their new roles, including breastfeeding, their newborn care, and they lacks time for themselves and their partners [2]. The physical sign and symptoms following childbirth such as exhaustion, back pain, perineal pain, dyspareunia, hemorrhoids, and urinary incontinence that influence on the mother’s health- related quality of life (HRQoL) [3]. The HRQoL is a comprehensive measures of health of physical, psychological, and social domains that are influenced by individual experiences, beliefs, expectations, and perceptions [4].

Globally, most maternal deaths and morbidities occur during the postpartum period. However, the burden is unequally high in Sub-Saharan African countries [5, 6]. In Ethiopia, 70% of maternal deaths occurred during the postpartum period [7].

It is evident that quality postnatal care services contribute to increase the postpartum quality of life. Despite the substantial benefit of postnatal care (PNC) in promotion of health, the service utilization is still low in Ethiopia [8]. A pooled estimate of PNC service utilization in Ethiopia was 32% [9].

Despite the health seeking behavior of the pregnant women has been improved and maternal health services ameliorated in Ethiopia. However, the HRQoL among postpartum women remain overlooked. In addition, postpartum quality of life lacks a precise definition and little is recognized about how HRQoL aspects vary in the first six weeks following childbirth. Even though assessing HRQoL is a key input for health planners and decision-makers, the existing knowledge in Ethiopia reveals that the elements that impact postpartum HRQoL in a local context and culture, but they haven’t emphasized the aspects specific to postpartum women, suggesting a paucity of evidence that could inform decision-making [10]. Therefore, the aimed of this study was to assess the health-related quality of life and associated factors among postpartum women in the Dendi district, West Shoa Zone, Oromia Region, Ethiopia.

## Methods

### Study area and period

The study was conducted at Dendi district, Oromia Region, Ethiopia from 23 August 2022 to 16 November 2022. Dendi is a district with 37 rural and 2 urban kebeles (the smallest administrative unit) [11]. The Dendi district is located 83 km West of Addis Ababa, the capital city of Ethiopia. According to the Dendi district health office report [11], the district consists of 1 primary hospital, 7 health centers, and 39 health posts, which provide curative, preventive, and promotive health services for an estimated total population of 197,964. From the total population of the Dendi district, 43,809 were reproductive-age women [11].

### Study design

A community-based cross-sectional study was conducted in the Dendi district.

### Population

#### Source population

All postpartum women living in the Dendi district.

#### Study population

All postpartum women lived in randomly selected kebele of the Dendi district during the data collection period.

### Eligibility criteria

#### Inclusion criteria

All postpartum women who gave birth within six weeks, whether alive or stillbirth and lived in the Dendi district.

#### Exclusion criteria

Women with chronic medical diseases who cannot respond to the interview during data collection period and women who have lived in the study area for less than six months were excluded from the study.

### Sample size and sampling techniques

#### Sample size determination

The sample size was calculated using the single population formula with the following assumptions: level of confidence was 95%, (Zα/2) = 1.96, marginal of sampling error tolerated (d) = 0.05 and *p* = 62.3% the prevalence of postpartum women with lower HRQoL from a similar study finding in Arba Minch town, Ethiopia [10]. Therefore, the sample size was determined as follows:

$$n\, = \,{{{{\left( {{{\rm{Z}}_{{\raise0.7ex\hbox{${\rm{\alpha }}$} \!\mathord{\left/{\vphantom {{\rm{\alpha }} 2}}\right.\kern-\nulldelimiterspace}\!\lower0.7ex\hbox{$2$}}}}} \right)}^2} \times {\rm{p}}\left( {1 - {\rm{p}}} \right)} \over {{{\rm{d}}^2}}}\, = \,{{{{\left( {1.96} \right)}^2}\, \times \,0.623\left( {1 - 0.623} \right)} \over {{{\left( {0.05} \right)}^2}}}\, = \,361$$

Since the total number of postpartum mothers who gave birth in the past six weeks in the Dendi district was 1,164 (less than 10,000), the population correction formula was used.

$\text{n}\text{f}=\frac{\text{n}}{(1+\frac{\text{n}-1}{\text{N}})}$ Where: n = calculated sample size (361)

nf = the required sample size for the targeted population (276).

Finally, after using the population correction formula, considering 10% as a non-response rate and a design effect of 1.5, the final sample size was **456.**

### Sampling techniques

A multi-stage stratified sampling procedure was used to select the sampling units. Firstly, the district was stratified into rural and urban Kebeles. Then, the 15 rural and 1 urban kebeles were selected from 39 kebeles by using lottery method. Secondly, the enumeration of those women who gave birth in the last six weeks was conducted in the selected kebeles in collaboration with the health extension worker. From this the lists of those women who gave birth in the last six weeks before data collection period and who lived in the study area at least for six months was prepared as a sampling frame. Finally, the study participants were selected using a computer generated simple random sampling technique from the lists of postpartum women in the selected kebeles using proportional allocation to the size of postpartum women along with their full addresses. The local extension worker assisted the data collectors in tracing the residences of the selected postpartum women.

### Study variables

#### Dependent variable

Health-related quality of life.

#### Independent variables

The independent variables include socio-demographic characteristics (mother’s age, mother’s educational status, marital status, occupation, husband’s educational status, household wealth index, baby care assistance), obstetrics and maternal health service-related factors (antenatal care utilization, mode of delivery, birth outcome, parity, newborn sex, place of delivery, postnatal care utilization), social support and postpartum depression.

### Operational definitions

The health-related quality of life was measured by scale to be in consolidation with the 36-Item Short Form Health Survey (SF-36) [12]. The scoring of HRQoL was measured by employing validated Persian sort HRQoL SF-36. The scoring of SF-36 was done according to the SF-36 Health Survey Manual and Interpretation Guide, and the following steps were followed to obtain the final score for each study subject [13]. The reverse coding was done for ten items in the SF-36 questionnaire. After reverse coding ten items, a raw score was computed for each domain by a simple algebraic sum of responses for all items in each HRQoL domain. Then each raw scale score was transformed from 0 to 100 (0–100 scale) by using the formula of transformed scale. Scores between these values represent the percentage of the total possible score achieved in each domain by the study subject. The final HRQoL mean score of SF-36 is obtained by calculating the mean of all the domain scores of each study subject.

$${\bf{Transformed}}{\rm{ }}\,{\bf{scale}}{\rm{ }} = {{\left( {actual\,score - lowest\,possible\,score} \right)} \over {\left( {possible\,raw\,score\,range} \right)}}*{\rm{ }}{\bf{100}}$$

Physical HRQoL (PCS) means was computed from physical functioning, bodily pain, role physical, and general health domain transformed score. Mental HRQoL (MCS) means was computed from mental health, social functioning, vitality, and role emotional domain transformed score. The HRQoL mean score was computed from the eight domains transformed scores. Using the standardized mean score of 50, the overall HRQoL, physical HRQoL, and mental HRQoL were dichotomized into higher and lower HRQoL.

**The Physical Component Summary (PCS)** mean score is computed as the arithmetic average of the transformed scores of bodily pain, physical functioning, general health, and physical role domains [13].

**The Mental Component Summary (MCS)** mean score is computed as the arithmetic average of the transformed scores of mental health, emotional role, social functioning, and vitality domains [13].

**Health-related quality of life (HRQoL)** mean score is computed as the arithmetic average of the transformed score of the eight domains of health-related quality of life (physical function, role limitations due to physical problems, bodily pain, general health, vitality, social function, role limitation due to emotional problems, and perceived mental health [12]. Then it was categorized as high when study participants scored greater than or equal to fifty and low when they scored less than fifty [14].

#### Postpartum depression

According to the Edinburgh postnatal depression scale, postpartum women who scored ≥ 13 are considered to have postpartum depression, and when postpartum women who scored < 13 are considered as not depressed [15].

#### Social support

The Oslo 3-Item Social Support Scale (OSSS-3) sum score can be categorized into three broad categories of social support. 3–8 (poor social support), 9–11 (moderate social support), and 12–14 [16].

#### Household asset

The income was determined using information obtained from the participant’s asset. Each asset was dichotomized as 1 if they had or 0 they had not.

### Data collection tool and techniques

Data was collected using a structured questionnaire and checklist adopted from a standard Medical Outcome Study (MOS) 36-item SF-36 health survey, Oslo 3-Item Social Support Scale, Edinburgh Postnatal Depression Scale (EPDS), and related literatures [3, 10, 16–18].

The questionnaire has five parts such as the socio-demographic characteristics, maternal health services utilization-related characteristics, postpartum depression assessment, health-related quality of life, and perceived social support. The MOS SF-36 was validated and translated for Ethiopia as an appropriate tool for measuring HRQoL in various population groups [19] and a well-known generic instrument, which has proved to be highly feasible and reliable and is a good choice to measure HRQoL among postpartum women [3, 10, 20, 21]. The medical outcome study of SF-36 consists of 36 questions and measures eight health-related domains, including physical functioning, physical role limitation (restraints on work or other regular daily activities due to physical health issues), bodily pain, general health perceptions (self-rated health), vitality, and social functioning (restraints in usual daily social interactions with family, friends, neighbors, or burial services), and perceived mental health (how the woman feels and how things have been for her). The scale scores within each domain ranged from 0 (corresponding to the worst possible state) to 100 (corresponding to the best possible state). The eight domains comprise two summaries: physical component summary (PCS) and mental component summary (MCS) [22].

The Edinburgh postnatal depression scale (EPDS) was used to assess postpartum women at risk of postpartum depression. This questionnaire includes ten items concerning common symptoms of depression, scored from 0 up to 3 (higher score indicating more depressive symptoms), and the total sum of the score is 30. A mother checks one of four possible answers closest to how she has felt during the past seven days [7].

The Oslo 3-Item Social Support Scale (OSSS-3) questionnaire was used to assess perceived social support. It consists of three questions that ask about the number of close friends, the degree of concern from others, and the relationship with neighbors, emphasizing the availability of practical help. The sum score ranges from 3 to 14 [16].

Household wealth status was assessed using 26 items (ownership of household durable asset data) for urban and rural residents adapted from the Ethiopian Demographic and Health Survey (EDHS) wealth index assessment questionnaire [18].

### Data collection techniques

Structured and face-to-face interviewer-administered questionnaires were used to collect the data from study participants. Four data collectors (BSc in midwifery) and two supervisors (with a BSc in public health) who are fluent in the Afan Oromo language were hired for data collection. All the interviewer-administered questionnaires took place in private spaces.

### Data quality assurance

Structured questionnaires were prepared in English, translated into Afaan Oromo (local language), and then translated back into English to check for consistency and correctness. Maximum efforts were made to minimize potential bias through: proper designing of the validated tools, training was given for data collectors and supervisors for one day about the objectives of the study; how to keep the confidentiality of the participants’ information; the contents of the questionnaire; filling out data collection formats, and data quality management by the investigator. Names or any other identifying information of the individual participant remains unknown to any person. The collected information was kept confidential by data collectors and investigators.

The questionnaire was pretested on 23 postpartum women who are living outside the study area but were not involved in the actual study. Cronbach’s alpha coefficient was used to ensure the reliability of the tools [23] and was found to be 0.87. Internal validity was ensured by measuring content validity ratio and was 0.2. Based on the pretest results, necessary modifications and corrections were made. Furthermore, the principal investigator and supervisors have made spot-checking and reviewed all the completed questionnaires daily during the data collection period to ensure completeness and consistency.

### Data processing and analysis

The collected data were entered into Epi Data version 3.1 and exported to Statistical package for social science version 26 for analysis. Data were cleaned by running the frequency and crosschecking any missing variables from the hard copy. Descriptive statistics such as mean, standard deviation, frequency, and percentage were used to summarize descriptive data. The mean and standard deviation were used to describe the health-related quality of life. The findings were presented as narratives, cross-tabulations, tables, and graphs. Binary logistic regression analysis was employed to determine factors associated with HRQoL. After testing the assumptions of binary logistic regression, variables with a p-value < 0.25 at the binary logistic regression analysis were considered candidates for multiple logistic regression analysis.

In the multivariable logistic regression model, a variable with a p-value < 0.05 was considered significantly associated variables with the outcome variable (HRQoL). Adjusted odds ratios (AOR) and 95% confidence intervals (CI) were calculated to measure the association’s strength between the overall HRQOL and independent variables. The researchers have controlled the effects of confounders with multivariable logistic regression analysis. The fitness of the final model was tested by Hosmer Lemeshow’s goodness of fit test and the model is a good fit to the data (*p* = 0.64). Multicollinearity among independently associated variables was checked by the multicollinearity diagnostic test, variance inflation factor (VIF). However, there were no identified variables with multicollinearity issues.

## Results

### Socio‑demographic and economic characteristics of study participants

From 456 samples, 429 were responded to the questionnaires with response rate 94.07%. As a result, 27 pieces of data were found as a missing data and the authors disregarded them from the final report. Out of the 429 samples, 267 (62.2%), were found in the age group 25–34 years with a mean age of 28.3 years and a standard deviation (SD) of 5.39. About 385 (89.7%) were rural area residents. Regarding educational status, 157 (36.6%) of the postpartum women had secondary school education. About household asset, 151 (35.2%) had lowest asset.

#### Reproductive, obstetrics, and maternal health services utilization-related characteristics of study participants

Based on the obstetrics and maternal health-services, 399 (93%), were multiparous, while 30 (7.0%) were primiparous. One-thirds 179 (41.7%) of the study participants had 3–4 children. About place of birth, 234 (54.5%) women gave birth at the health facility and 331 (77.2%) had received at least one PNC. Furthermore, 23 (5.4%) experienced obstetric complications during pregnancy, 61 (14.2%) reported having difficulty during delivery, and 14 (3.3%) had complications after giving birth (Table 1).

**Table 1:** Frequency distribution of reproductive, obstetrics, and maternal health services utilization-related characteristics of the study participants (*n* = 429) in Dendi district, West Shoa Zone, Oromia Region, Ethiopia, 2022

Respondents characteristics	Categories	Frequency	Percent
Parity	Primiparous Multiparous	30 399	7.0 93.0
Number of living children	1–2 3–4 ≥ 5	80 179 170	18.6 41.7 39.6
State of last pregnancy	Planned Unplanned	277 152	64.6 35.4
Place of birth	Home Health center Hospital	73 234 122	17.0 54.5 28.4
Mode of delivery	Spontaneous vaginal delivery Episiotomy Cesarean section	308 61 60	71.8 14.2 14.0
Birth outcome	Alive Dead	424 5	98.8 1.2
Newborn sex	Male Female	209 220	48.7 51.3
Complication during pregnancy	Yes No	23 406	5.4 94.6
Complication during delivery	Yes No	61 368	14.2 85.8
Complication after delivery	Yes No	14 415	3.3 96.7
Hospital admission history of newborn	Yes No	8 421	1.9 98.1
Received at least four ANC visits	Yes No	151 278	35.2 64.8
Received at least one PNC	Yes No	331 98	77.2 22.8

#### Postpartum depression and related psychosocial characteristics of study participants

The OSSS-3 scale found that, more than one-third of study participants 185 (43.1%) were with poor social support, followed by moderate social support 179 (41.7%), and strong social support, 65(15.2%). Nearly three-fourths, 320 (74.6%) had no baby care assistant. About one-fourth109 (25.4%) had postpartum depression.

#### Health-related quality-of-life (HRQoL) of the participants

**Fig. 1:**
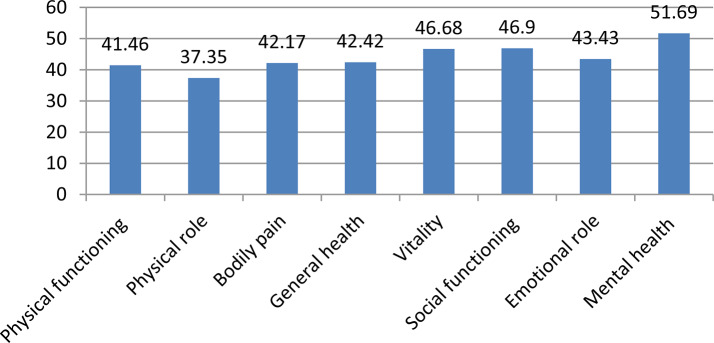
Distribution of mean score on eight subscales of SF-36 health-related quality of life in Dendi district, West Shoa Zone, Oromia Region, Ethiopia, 2022

**Fig. 2:**
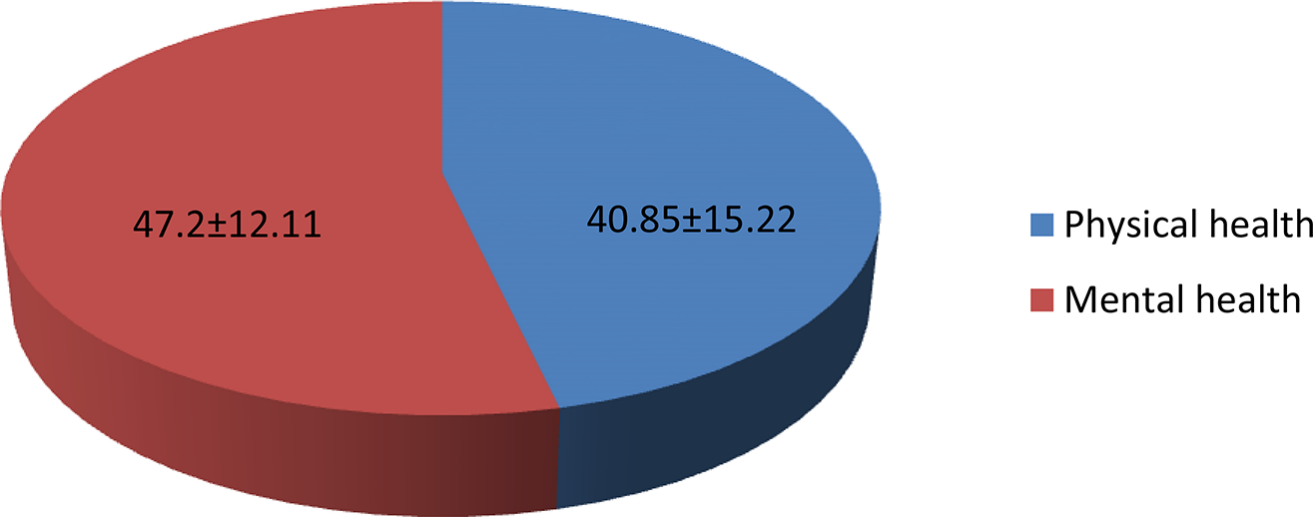
Distribution of mean score of SF-36 health-related quality of life two-component summary score with 95% CI in Dendi district, West Shoa Zone, Oromia Region, Ethiopia, 2022

The SF-36 scale found that three-fourths 316 (73.7%) (95% CI: 69.4, 77.7) of participants had a low health-related quality of life. The mean of HRQoL of sample was 44.02 ± 10.4 (95% CI: 43.03, 45.01. From the eight domains, the lowest mean score was observed in the role physical dimension with a Mean ± SD of 37.35 ± 14.2, whereas the highest mean score in the mental health dimension was 51.69 ± 22.98 (Fig. 1). Moreover, the mean of mental component summary (MCS) score was high (47.20 ± 12.11) when compared with the mean of physical component summary (PCS) score of 40.85 ± 15.22 (Fig. 2).

### Factors associated with health-related quality of life

In this study, mode of delivery, place of residence, mother’s level of education, husband’s employment status, baby care assistance, pregnancy planning status, newborn sex, receiving at least four antenatal visits, receiving postnatal care, perceived social support, postpartum depression, age, complications during the delivery, and household asset were identified as a candidate variables for multivariable logistic regression analysis. The multivariable logistic regression analysis showed that place of residence, women’s level of education, receiving at least four antenatal visits, at least one postnatal care, social support, postpartum depression, cesarean section delivery, and household asset were significantly associated with low health-related quality of life.

The study showed that postpartum women who had no education were 3.44 times more likely to have a low health-related quality of life than those postpartum women who had education [AOR = 3.44, 95% CI: (1.35–8.74)]. Postpartum women who were delivered by cesarean section were 3.18 times more likely to have a low health-related quality of life than postpartum women who give birth by vaginal delivery [AOR = 3.18, 95% CI: (1.09–9.26)]. This study also identified that postpartum women with poor perceived social support were 2.23 times more likely to have a low health-related quality of life than postpartum women with strong social support [AOR = 2.23, 95% CI: (1.02–4.89)]. Postpartum women who had postpartum depression were 2.99 times more likely to have a low health-related quality of life than postpartum women who had no postpartum depression [AOR = 2.99, 95% CI: (1.52–5.86)].

Additionally, postpartum women with the lowest household asset were 5.68 times more likely to have a low health-related quality of life compared to those with highest household asset [AOR = 5.68, 95% CI: (2.74–11.76)]. Postpartum women who had received at least 4 ANC for the last pregnancy were 44% less likely to have a low health-related quality of life compared to those who had not received at least 4 ANC for the previous pregnancy [AOR = 0.56, 95% CI: (0.33–0.95)]. Postpartum women who received at least one postnatal care were 70% protective for low health-related quality of life compared to those who had not received any postnatal care [AOR = 0.30, 95% CI: (0.14–0.62)] (Table 2).

**Table 2:** Bivariate and multivariate logistic regression analysis for factors associated with health-related quality of life among postpartum women (*n* = 429) in Dendi district, West Shoa Zone, Oromia Region, Ethiopia, 2022

Variables	Categories	HRQoL		COR(95% CI)	P value	AOR(95% CI)	P value
		Low	High				
**Age of respondents**	17–24	72	34	0.51 (0.23–1.12)	0.096	0.50 (0.19–1.26)	0.145
	25–34	199	68	0.71 (0.35–1.46)	0.358	0.81 (0.34–1.89)	0.630
	35–45	45	11	1		1	
**Mothers education**	No formal education	18	113	3.24 (1.46–7.2)	0.004	3.44 (1.35–8.74)	0.009*
	Primary	24	73	1.57(0.72–3.41)	0.252	1.49 (0.59–3.76)	
	Secondary	56	101	0.93(0.46–1.88)	0.847	0.48 (0.20–1.15)	0.398
	Above secondary	15	29	1		1	0.102
**Husband occupation**	Daily laborers	12	1	4.38 (0.56–34.2)	0.159	4.72 (0.46–4.84)	0.191
	Gov’t employee	39	14	1.01 (0.56–1.96)	0.959	1.31 (0.56–3.09)	0.526
	Private employee	35	12	1.06 (0.52–2.14)	0.860	1.36 (0.49–3.78)	0.553
	Farmer	230	84	1		1	
**Residence**	Urban	27	17	0.52 (0.27–1.01)	0.054	0.27 (0.10–0.74)	0.011*
	Rural	289	96	1		1	
**Baby care assistance**	Yes No	73 243	36 77	0.64 (0.40–1.03) 1	0.068	0.90 (0.49–1.64) 1	0.744
**Household asset**	Lowest	132	19	3.08 (1.69–5.613)	0.001	5.68 (2.74–11.76)	0.001*
	Middle	87	51	0.75 (0.45–1.24)	0.272	1	0.764
	Highest	97	43	1		0.91 (0.50–1.65)	
**Perceived** **social support**	Poor	151	34	2.43 (1.29–4.567)	0.006	2.23 (1.02–4.89)	0.043*
	Moderate	123	56	1.20 (0.66–2.18)	0.545	0.94 (0.44–1.98)	0.882
	Strong	42	23	1		1	
**Postpartum depression**	Depressed Not depressed	92 224	17 96	2.319 (1.31–4.10) 1	0.004	2.99 (1.52–5.86) 1	0.001*
**Mode of delivery**	Cesarean section	55	5	4.55 (1.77–11.68)	0.002	3.18(1.09–9.26)	0.034*
	Vaginal delivery	261	108	1		1	
**Newborn sex**	Male	160	49	1.34 (0.86–2.06)	0.185	1.11 (0.66–1.88)	0.678
	Female	156	64	1		1	
**Complication after delivery**	Yes	10	4	0.89 (0.27, 2.89)	0.84	2.32 (0.91–5.89)	0.077
	No	306	109	1		1	
**Complication during delivery**	Yes	53	8	2.64 (1.21–5.75)	0.014	2.32 (0.91–5.89)	0.077
	No	263	105	1		1	
**ANC visits ≥ 4**	Yes	104	47	0.68 (0.44–1.07)	0.098	0.56 (0.33–0.95)	0.032*
	No	212	66			1	
**Received at least one PNC**	Yes	235	96	0.51 (0.28–0.91)	0.023	0.30 (0.14–0.62)	0.001*
	No	81	17	1		1	
**Pregnancy state**	Planned	198	79	0.72 (0.45–1.14)	0.167	0.62 (0.35–1.08)	0.092
	Unplanned	118	34	1		1	

*Significant at P value < 0.05

COR: crude odds ratio; AOR: adjusted odds ratio; CI: confidence interval

1: Reference group

## Discussion

During the postnatal period, the women’s health-related quality of life is determined by medical, psychological and social factors. The study revealed that residence, education, at least four ANC contact, receiving at least one PNC, poor social support, postpartum depression, cesarean delivery and lowest household asset were found to have significantly associated with low health-related quality of life among postpartum women. The study demonstrated that cesarean delivery affected the health-related quality of life postpartum women. This finding is consistent with previous studies [24–28]. The possible association may be physical functioning probably less for women who underwent cesarean section than vaginal delivery. Lack of social support contributes to low health-related quality of life of postpartum women. This findings is congruent with a study conducted in Rwanda [29], Turkey [30], and Japan [31]. This is may be due to the fact that low perceived social support exacerbates postpartum depression, psychological distress, and poor mental health status. The finding of this study indicated that postpartum depression significantly affects the HRQoL of postpartum women. This finding is consistent with previous studies [10, 17, 28, 32–38]. The possible explanation might be due to depression affects mental health as well as it affects the women’s ability to function, positive interpersonal relationships, sleep patterns, and social engagement [39].

The result of this study revealed that women who utilized ANC visits and PNC positively associated with high health-related quality of life among postpartum women. The finding of this study is in line with a study conducted in Bangladesh [26], Rwanda [29] and Arba Minch, Ethiopia [10]. This may be due to the fact that continuum maternal health services have become a key component to promoting the health and well-being of postpartum women.

Finding in this study indicates that health-related quality of life among postpartum considerably determined by residence. It argues that living in urban had positive effect on high health-related quality of life of postpartum women. In contrast to this finding, a study conducted in Malawi [29] unfold that living in urban embodied lower HRQoL than postpartum women living in rural. Regarding the disparities in quality of life of postpartum women between rural and urban areas, there is no agreement in the existing investigation.

The results showed that women had no education were more likely to have low health-related quality of life than their counterparts. This finding is consistent with previous studies [10, 40]. The possible explanation might be women having no education are less likely to attend maternal care health services, inability to comprehend obstetric complications and delay to seek healthcare immediately from skilled healthcare workers.

## Limitations of the study

Health-related quality of life, perceived social support and postpartum depression were measured based on the respondents’ self-report which may prone to response set bias. In addition, since some of the existing knowledge of literatures were conducted using a different tool that has not been validated in Ethiopia, this could results in the variation of the measurements, which limits us from further comparing the current study’s findings with other studies.

## Conclusions

The health-related quality of life among postpartum women was very low. The study found that education, urban residing, received at least four ANC contact, received at least one PNC, good social support, highest household asset and vaginal delivery were significantly associated with increased odds of high health-related quality of life. Postpartum women with low socio-economic status and inadequate maternal health services utilization were associated with a low health-related quality of life. Therefore, the study implies a robust emphasis is needed to improve the women’s socio-economic status and maternal health services like PNC to increase the health-related quality of life of postpartum women.

## Data Availability

Data are available on the reasonable request from the corresponding author.

## Abbreviations

ANC: Antenatal Care
AOR: Adjusted Odds Ratio
COR: Crude Odds Ratio
CI: Confidence Interval
EPDS: Edinburgh Postnatal Depression Scale
ERB: Ethical Review Board
HRQoL: Health-Related Quality of Life
HSTP: Health Sector Transformation Plan
MMR: Maternal Mortality Ratio
MCS: Mental Component Summary
NVD: Normal Vaginal Delivery
OSSS-3: Oslo Social Support Scale 3-Item
PCS: Physical Component Summary
PNC: Postnatal Care
QoL: Quality of Life
SD: Standard Deviation
SF-36: 36-Item Short Form Health Survey
WHO: World Health Organization
